# Omega-3 Source Matters: Comparative Lipid Signatures and Quantitative Distribution of EPA/DHA Across Marine Resources

**DOI:** 10.3390/md24010004

**Published:** 2025-12-20

**Authors:** Kolos Makay, Carola Griehl, Stephan Schilling, Claudia Grewe

**Affiliations:** 1 Doctoral Center Life Sciences, Research Center and Graduate School of Anhalt University of Applied Sciences, Bernburger Str. 55, 06366 Köthen, Germany; kolos.makay@hs-anhalt.de (K.M.);; 2 Competence Center Algal Biotechnology, Bernburger Str. 55, 06366 Köthen, Germany; 3 Fraunhofer Institute for Cell Therapy and Immunology, Weinbergweg 22, 06120 Halle (Saale), Germany

**Keywords:** EPA, DHA, microalgae, krill, fish, *Schizochytrium*, HPTLC-GC-MS, marine lipids

## Abstract

Eicosapentaenoic acid (EPA) and docosahexaenoic acid (DHA) are essential omega-3 polyunsaturated fatty acids (n-3 PUFAs) with well-established health benefits. They occur primarily in marine resources, while their quantitative distribution within the glycerolipidome is rarely analyzed. Therefore, we investigated major commercial sources, including 12 microalgal species, the protist *Schizochytrium* sp., four fish species, and nine commercial n-3 supplements (fish, krill and *Schizochytrium*-derived “algal” oils) by high-performance thin-layer chromatography–gas chromatography–mass spectrometry (HPTLC–GC–MS). The class-resolved mapping of EPA and DHA revealed signature lipid profiles across all sources. In microalgae, 60–80% of EPA was localized in glycolipids, whereas in *Schizochytrium* and fish, >90% of DHA occurred in triacylglycerols. Krill oils exhibited phospholipid-rich profiles with ~70% of phosphatidylcholine-bound DHA. Nutritional indices also highlighted major differences: fish and fish oils showed favorable PUFA-to-saturated FA ratios (>0.45) and hypocholesterolemic-to-hypercholesterolemic ratios (>1), while *Schizochytrium*-based “algal” oils even surpassed these values. The microalgae *Nannochloropsis granulata* contained the highest EPA content in biomass form, combined with favorable nutritional indices. Beyond total n-3 content in relation to recommended daily intake values, the lipid-class distribution and nutritional indices should be considered decisive metrics for evaluating the health relevance of n-3 resources in the human diet.

## 1. Introduction

Omega-3 (n-3) long-chain polyunsaturated fatty acids (LC-PUFAs), such as eicosapentaenoic acid (EPA; C20:5 n-3) and docosahexaenoic acid (DHA; C22:6 n-3), play critical roles in human health. In addition to serving structural functions within cellular membranes, EPA and DHA modulate endogenous antioxidant defense, exert potent anti-inflammatory effects, thereby promoting cardiovascular and metabolic health [1,2,3]. More recently, studies have also investigated the role of n-3 LC-PUFAs in protein misfolding disorders, including their effects on membrane microenvironments, amyloid aggregation, and synaptic integrity [4,5,6]. In line with these mechanisms, n-3 supplementation has been shown to attenuate amyloid and α-synuclein pathology in animal models, while clinical studies suggest potential—though stage- and genotype-dependent—benefits in Alzheimer’s disease [7,8,9].

Reflecting the physiological importance of EPA and DHA, global health authorities recommend a daily intake of 250–500 mg combined EPA DHA for individuals without underlying health conditions, primarily to support general health and reduce long-term disease risk [10]. Traditionally, the primary dietary sources of EPA and DHA have included fish (e.g., salmon, sardines, anchovies), shellfish and crustaceans. However, meeting the recommended daily intake (RDI) demand through seafood alone is challenging due to dietary preferences and access to marine sources. Consequently, fish and krill oil supplements have become widespread commercial sources of n-3 LC-PUFAs [11,12]. This increasing reliance on seafood-derived oils, however, is straining marine ecosystems and contributing to overfishing [13,14]. Consequently, growing sustainability concerns have prompted additional research directions, particularly those targeting the de novo producers at the base of the marine food web—photosynthetic microalgae and heterotrophic protists such as *Schizochytrium* spp. These microorganisms can be cultivated on non-arable land under controlled conditions, typically in bioreactors, thus enabling reliable production while also reducing pressure on marine ecosystems. Despite the associated higher production costs, microalgal and protist-derived oils have nevertheless emerged and entered the market as viable alternatives to traditional fish- and krill-based sources [15,16,17].

Research in marine biotechnology leveraging microalgae and marine protists has predominantly aimed at increasing EPA and DHA contents and productivities to deliver non-fish alternatives capable of sustaining supply in the context of growing global demand. By contrast, comparatively little consideration has been given to the chemical forms in which these fatty acids (FAs) occur. Rather than existing as free fatty acids (FFAs), EPA and DHA are typically esterified into specific glycerolipids. In marine sources, these glycerolipid subclasses include the phospholipids: phosphatidylcholine (PC), phosphatidylethanolamine (PE), phosphatidylinositol (PI), phosphatidylserine (PS), and phosphatidylglycerol (PG), the neutral lipids such as triacylglycerols (TAG), diacylglycerol (DAG), monoacylglycerol (MAG); algae specific glycolipids; monogalactosyldiacylglycerol (MGDG) and digalactosyldiacylglycerol (DGDG); the sulfolipid sulfoquinovosyldiacylglycerol (SQDG); and also algae specific betaine lipids (BL) [18,19,20]. Beyond the chemical structures, different lipid classes significantly influence bioavailability, digestion, and physiological effects. For instance, n-3 LC-PUFAs esterified to phospholipids have been reported to exhibit different digestibility and tissue uptake compared to those stored in neutral lipids, such as TAG [21]. These structural and compositional differences underscore that EPA and DHA are not functionally equivalent across different sources. From a nutritional standpoint, it is therefore essential to assess not only the total amounts of EPA and DHA but also the specific lipid classes in which they are incorporated, along with their quantitative distribution [19,22].

Gas chromatography–mass spectrometry (GC-MS) of fatty acid methyl esters (FAMEs) remains a widely used and robust quantitative approach for determining overall FA profiles. However, because GC-based workflows require prior transesterification, they do not retain information on the intact lipid molecular species from which FAs originate. Complementary methods that preserve lipid class structure—such as solid-phase extraction coupled with GC-MS—have improved class-level resolution but still provide limited insight into the specific distribution of EPA and DHA among individual glycerolipid species [23]. Consequently, ultra-high-performance liquid chromatography (UHPLC) coupled with mass spectrometry has become widely used in lipidomics, enabling detailed profiling and structural characterization of intact glycerolipid species. However, challenges such as variable ionization efficiencies and the limited availability of structurally matched internal standards continue to influence the accuracy of absolute quantification [24]. To address current analytical challenges and complement existing lipidomic workflows, high-performance thin-layer chromatography (HPTLC) has gained renewed attention as a robust tool in the field of lipidomics [25,26]. For instance, HPTLC has been employed to quantify polar and neutral lipids in mammalian cells, as well as to assess lipid class–resolved FA distributions in microalgae [20,27].

Building on these developments, we applied an HPTLC–GC–MS hyphenated strategy to systematically characterize a broad range of n-3 sources. To capture the diversity of n-3 LC-PUFA we selected representative species and product types spanning the major industrial sources of EPA and DHA. The twelve microalgal species include phylogenetically and functionally distinct taxa (Eustigmatophyceae, Bacillariophyceae, Prymnesiophyceae, Chlorodendrophyceae, and Porphyridiophyceae) chosen based on being commercially known n-3 LC-PUFA producers, thereby enabling a comparative assessment across divergent lipid architectures. Three *Phaeodactylum tricornutum* strains were included because they represent the most widely cultivated diatom, yet differ in growth history, cultivation origin, providing a controlled test case for both strain-level and cultivation condition (e.g., temperature, light intensity and regime) variations, which are known to affect lipid class distribution. The four oily fish species were selected as globally consumed marine sources that differ in trophic level, metabolic strategy, and lipid storage patterns, enabling evaluation of how ecological and physiological traits influence EPA and DHA partitioning between structural and storage lipids. Finally, the three n-3 supplement types—fish oil, krill oil, and microbial oil—were chosen because they represent the dominant commercial formulations available to consumers, each produced through distinct extraction and refining workflows that generate divergent lipid class profiles. Together, this structured selection provides a biologically and industrially relevant cross-section of marine n-3 LC-PUFA sources, forming a rational basis for applying our lipid-class–resolved quantification workflow.

This integrative HPTLC–GC–MS workflow enabled the quantification of both total FAs and lipid classes, as well as the absolute, class-resolved quantification of EPA and DHA across 13 distinct lipid classes, thereby addressing limitations in previously underrepresented aspects of n-3 lipid analysis. Applied to oily fish, commercial supplements, and emerging microbial alternatives, the method generated detailed lipid fingerprints that revealed distinct, source-dependent lipid signatures. Furthermore, incorporating nutritional and health indices, including the index of atherogenicity (IA), index of thrombogenicity (IT), and hypocholesterolemic/hypercholesterolemic ratio (h/H), extended the analysis beyond compositional data, linking lipid class distribution to metabolic and cardioprotective potential. Collectively, these findings advance understanding beyond bulk EPA and DHA measurements by providing deeper resolution of marine glycerolipidome diversity across marine n-3 sources, which could guide future investigations into bioavailability, functionality, and potential health applications.

## 2. Results and Discussion

### 2.1. Overall EPA, DHA and Other Signature FA Contents

**Figure 1:**
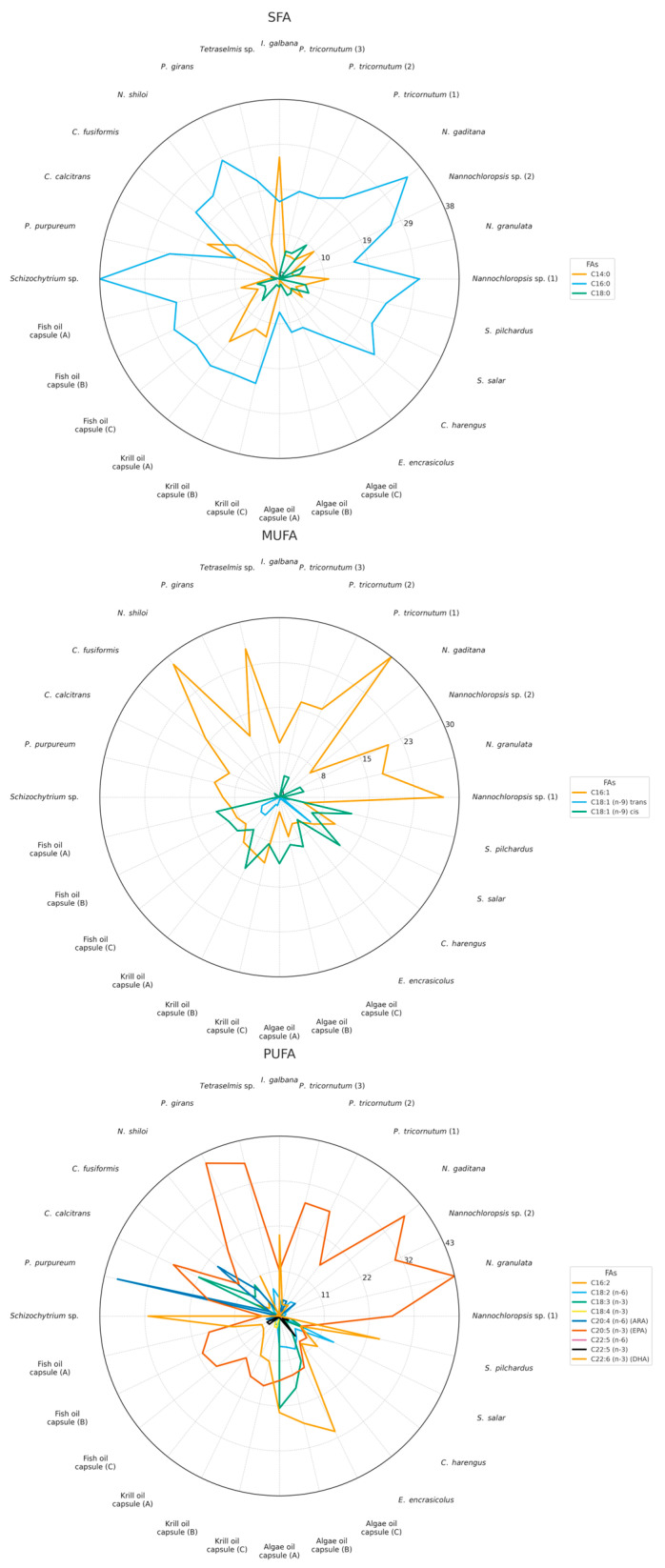
The Relative distribution of individual fatty acids (FAs) expressed as a percentage of total FA content. Saturated fatty acids (SFAs) are shown first, followed by monounsaturated fatty acids (MUFAs) and polyunsaturated fatty acids (PUFAs). Only FAs contributing more than 5% of the overall FA content in any of the sources are displayed. Minor FAs can be found in Supplementary Figure S2.

In this study, we analyzed a comprehensive dataset of FA profiles from 28 individual n-3 LC-PUFA sources. The study encompassed five distinct sample groups: 12 microalgal species, one marine heterotrophic protist (*Schizochytrium* sp.), four marine fish species, and nine commercial oil supplements from fish, krill and *Schizochytrium* (labeled as “algal” oil). For each sample, the absolute contents of individual FAs (expressed as mg g^−1^ dry weight (DW), mean ± SD) are provided in the Supplementary Table S1, enabling detailed comparison of FA profiles across taxa and product types, while the relative % of predominant FAs are presented in Figure 1, and minor FAs are represented in Supplementary Figure S2.

Out of 28 samples examined in total, only five revealed an EPA proportion of over 30%, and only two of these exceeded 40% of EPA in total FA content. All of these samples belong to the group of microalgae. Among the microalgae examined, EPA was the predominant n-3 LC-PUFA, whereas only seven of the microalgae examined produced DHA, and only in limited amounts. It is noticeable that the EPA proportion of 40% of total FAs is exceeded only by *P. gyrans* and *N. granulata.* Notably, all four *Nannochloropsis* species analyzed, along with *P. tricornutum* (2) and *P. tricornutum* (3), exhibited the highest EPA content among the microalgae species, although there were significant differences within the same genus and even within species (*p* < 0.01). For instance, *N. granulata* and *N. gaditana* contained 51.0 ± 2.25 mg g^−1^ and 36.08 ± 2.59 mg g^−1^ EPA, respectively, accounting for approximately 43.0 ± 1.90 and 38.33 ± 2.75% of their total FA content (Figure 1). These findings align with previous reports and highlight the typical FA profile of the *Nannochloropsis* genus, which produces EPA but lacks the enzymes required for DHA synthesis, confirming its role as a source of EPA-rich biomass [28,29]. Similarly, two out of the three *P. tricornutum* biomasses contained substantial amounts of EPA, ranging from 31.85 ± 1.39 to 36.58 ± 2.20 mg g^−1^ DW. In contrast, DHA levels remained relatively consistent across all three *P. tricornutum* samples (*p* > 0.01), ranging from 3.37 ± 0.48 to 3.85 ± 0.36 mg g^−1^, constituting less than 5% of the total FAs, in good agreement with previous findings [30,31].

Although various factors, such as cultivation conditions, influence FA content and composition, the values observed in this study fall within ranges previously reported in the literature. Inherent genetic differences may also contribute to this variability; for example, a comparative study of *Nannochloropsis* and *Microchloropsis* (formerly classified under the *Nannochloropsis* genus) revealed significant discrepancies in EPA content, even under identical cultivation conditions, highlighting the combined impact of genetic background and environmental parameters on lipid biosynthesis [30,32].

In contrast to most algal species, which exhibited negligible levels of DHA, there were notable exceptions such as *I. galbana* and *P. gyrans* containing 14.6 ± 1.13 mg g^−1^ and 9.08 ± 0.65 mg g^−1^ DHA, respectively. Despite this, *P. gyrans* still exhibited EPA as its dominant n-3 LC-PUFA (34.8 ± 2.5 mg g^−1^), whereas *I. galbana* had a notably lower EPA content (8.2 ± 1.0 mg g^−1^), making DHA the principal n-3 LC-PUFA in this species. These results are consistent with previously published data, although they are slightly higher or lower in some instances, demonstrating the plasticity of the algal lipidome in response to different seasons, geographic latitude and/or photobioreactor type [33,34,35].

In contrast to microalgae and all other examined sources, DHA levels were exceptionally high in the heterotrophic protist *Schizochytrium* sp., which accumulated 142.1 ± 9.54 mg g^−1^ DHA, approximately 32% of total FAs (Figure 1), being consistent with previous reports [17,36]. In contrast, the EPA content in *Schizochytrium* sp. was substantially lower, at 18.31 ± 2.53 mg g^−1^, placing it at the lower end of the range observed across the algal biomasses in this study. This pronounced difference between DHA and EPA content reflects the organism’s known biosynthetic specialization. *Schizochytrium* synthesizes DHA via the polyketide synthase pathway but lacks the full set of desaturase and elongase enzymes required for efficient EPA synthesis [37].

Based on these observations, it is evident that no single microalgal species or *Schizochytrium* sp. studied can efficiently provide high levels of both EPA and DHA. Most phototrophic algae appear to be metabolically channeled toward EPA synthesis, with *I. galbana* being one of the few exceptions, showing modest DHA accumulation but still at relatively low absolute levels (14.46 ± 1.13 mg g^−1^). In contrast, marine protist species such as *Schizochytrium* are specialized in DHA production.

Among the fish species examined, a consistent pattern emerged, wherein the DHA content exceeded the EPA content in all cases. Anchovy exhibited the highest levels of both EPA (14.23 ± 0.98 mg g^−1^) and DHA (15.5 ± 2.05 mg g^−1^) on a dry matter basis, which is consistent with its classification as a small pelagic fish known for efficiently accumulating n-3 FAs [38,39]. Salmon and herring had slightly lower EPA (9.37 ± 0.93 and 10.85 ± 0.66 mg g^−1^) and moderate DHA (9.41 ± 1.73 and 15.11 ± 1.22 mg g^−1^) contents, respectively. Interestingly, sardines showed the highest DHA content among the four species (25.0 ± 3.16 mg g^−1^), whereas their EPA content (9.52 ± 0.52 mg g^−1^) fell within the range observed in salmon and herring. These FA contents, measured on a dry-weight basis, corresponded to approximately 0.23 ± 0.01% to 0.35 ± 0.02% EPA and 0.25 ± 0.03% to 0.62 ± 0.08% DHA, on a wet-weight basis. These values are considerably lower than those reported in the literature, which generally cites ranges of 0.5–1.5% EPA and 0.9–2.3% DHA by wet weight [40,41,42]. This discrepancy may be influenced by multiple factors, including methodological differences between studies, variations in sample handling and storage (e.g., oxidation in commercially sourced fish), and the limited sample size available for our analysis. Nonetheless, the lower values we observed remain consistent with reports suggesting a gradual decline in n-3 LC-PUFA levels—particularly EPA—in certain fish species over past decades [43], although this trend cannot be confirmed from our data alone. Furthermore, a major contributing factor is the shift in aquaculture feed formulations, with traditional marine-based feeds rich in n-3s increasingly being replaced by plant-based alternatives, which contain significantly lower amounts of LC-PUFAs [44]. Wild fish are also affected because climate-induced ocean warming alters the FA composition of primary producers, such as microalgae. Warmer temperatures have been associated with reduced PUFA synthesis in microalgae, thereby limiting EPA transfer through marine food webs to higher trophic levels, including fish [45].

As expected, the commercial oil supplements contained the highest overall levels of EPA and DHA. The three fish oil capsules had EPA contents ranging from 142.44 ± 5.83 to 176.49 ± 11.60 mg g^−1^, and DHA levels from 40.29 ± 4.10 to 94.27 ± 7.03 mg g^−1^. In contrast, the algal oil capsules, although derived from *Schizochytrium* sp., showed a DHA content between 178.82 ± 5.44 and 259.10 ± 3.22 mg g^−1^ and EPA levels ranging from 12.34 ± 1.86 to 119.48 ± 3.64 mg g^−1^. Krill oils were intermediate, showing EPA levels (91.22 ± 7.17 and 120.04 ± 6.08 mg g^−1^) more similar to algae oil and DHA levels (44.05 ± 3.60 to 78.17 ± 1.21 mg g^−1^) closer to fish oil. These values closely matched the manufacturers’ label claims, although the measured EPA and DHA contents were consistently 10 to18% lower, likely reflecting partial degradation during storage or slight overestimation in labeling, as reported previously [46].

The grouping of samples into fish species, fish oil capsules, krill oil capsules, algal oil capsules, *Schizochytrium* sp., and other microalgae revealed substantial differences in both FA composition and efficiency of EPA+DHA provision (Table 1). Fish species contained moderate levels of EPA (9–14 mg g^−1^) and DHA (9–25 mg g^−1^). Because fish is consumed as a food product, a 30–50 g serving (corresponding to approximately 7–13 g of dry tissue) is small in the context of a typical meal yet already provides enough EPA and DHA to meet the minimal RDI. Their FA profile was balanced across saturated fatty acid (SFA) (36–61 mg g^−1^), monounsaturated fatty acid (MUFA) (18–38 mg g^−1^), and PUFA (41–86 mg g^−1^), with a broad n-3/n-6 ratio (1.4–9.6:1). As expected, fish oil capsules were more concentrated, with EPA (142–176 mg g^−1^) and DHA levels sufficient to meet the RDI with about 1.1 g of oil. However, they also contained high SFA (324–350 mg g^−1^) but favorable n-3/n-6 ratios (≈6.4:1).

Interestingly, while DHA dominated in fish muscle tissue, fish oil supplements exhibited a higher EPA-to-DHA ratio. This pattern likely reflects that some fish species and tissues used as raw material—such as adipose tissue—naturally contain higher proportions of EPA than DHA [47,48]. Krill oil capsules contained less total EPA+DHA (135–198 mg g^−1^) compared to fish and algae oil capsules, covering the RDI with 1.3–1.9 g. Their FA profile included 280–360 mg g^−1^ SFA, 141–185 mg g^−1^ MUFA, and 187–258 mg g^−1^ PUFA. Their n-3/n-6 ratios (5.5–7.0:1) resembled those of fish oils, but the phospholipid-bound form of n-3 PUFAs was reported to be associated with improved absorption in some studies [21]. Algal oil capsules provided 291–378 mg g^−1^ EPA+DHA, dominated by DHA (179–259 mg g^−1^), and were the most efficient source in terms of the amount required to achieve the RDI, needing only 0.7–0.8 g. They also exhibited a highly PUFA-rich profile (529–567 mg g^−1^), low SFA and MUFA fractions, and the most favorable n-3/n-6 ratios (6.75–7.39:1). *Schizochytrium* sp. biomass also provided a DHA-dominant profile (~160 mg g^−1^ EPA+DHA), covering the RDI with ~1.6 g and showing a favorable n-3/n-6 ratio (≈3.8:1). The microalgae group was the most variable (8–66 mg g^−1^ EPA+DHA), requiring 5–21 g to meet the RDI. Their FA distributions (20–65 mg g^−1^ SFA, 6–43 mg g^−1^ MUFA, 33–68 mg g^−1^ PUFA) and n-3/n-6 ratios (0.56–15.6:1) were strongly species and strain-dependent.

Currently, dietary guidelines recommend EPA and DHA as a combined intake, reflecting their overlapping functions and natural co-occurrence in foods [49,50]. Yet exceptions exist: DHA is emphasized during pregnancy for neurodevelopment, while high-dose EPA is used in cardiovascular therapy. Our dataset mirrors this distinction: fish oils were EPA-rich, algal oils and *Schizochytrium* were DHA-dominant, and krill oil offered intermediate profiles. These compositional differences are meaningful because EPA and DHA are not interchangeable. EPA mainly reduces inflammation and platelet aggregation by altering eicosanoid synthesis, whereas DHA stabilizes membrane fluidity, supports neural signaling, and generates specialized pro-resolving mediators [51,52,53,54].

Overall, algal oil capsules provided DHA and PUFA levels, as well as n-3/n-6 ratios, that were comparable to those observed in krill and fish oil products (Table 1). Fish species provide naturally integrated proportions of EPA, DHA, and other PUFAs, accompanied by balanced n-3/n-6 ratios. *Schizochytrium* biomass offers a consistent DHA-focused profile suitable for vegan supplementation and infant nutrition. Other microalgal biomasses remain promising but require careful strain selection. From a nutritional perspective, algal-derived oils not only achieve RDI coverage with minimal intake but also counteract the excess of n-6 FAs typical of Western diets, supporting cardiovascular, cognitive, and anti-inflammatory health outcomes [49,53].

In addition to absolute quantification of EPA and DHA, relative compositional analysis (% of total FA) revealed distinct, species-specific FA signatures with both nutritional and taxonomic relevance (Figure 1), particularly with respect to palmitoleic acid (C16:1n-7), linoleic acid (LA, C18:2 n-6), α-linolenic acid (ALA, C18:3 n-3), and several LC-PUFAs, such as arachidonic acid (ARA, 20:4 n-6) and docosapentaenoic acid (DPA, C22:5).

Palmitoleic acid (16:1n-7) occurred at substantial levels in several of the microalgal samples analyzed, including most *Nannochloropsis* species—but not in *N. gaditana*—, *Phaeodactylum tricornutum*, *Tetraselmis* sp., *N. shiloi*. Fish oils and commercial oil supplements, by contrast, generally contained < 8% C16:1, with oleic acid (C18:1) being the dominant MUFA. In this context, C16:1 was markedly more prevalent in the algal samples than in the fish and commercial oils, a pattern also reflected in the spider-web profiles (Figure 1).

The distribution of LA and ALA further emphasized differences across sample types. Salmon contained elevated levels of LA (~14% of total FAs), likely reflecting the incorporation of plant-based oils in aquaculture feeds, as reported previously [44]. Algal oil supplements, in turn, exhibited high ALA contents of up to 10%, consistent with the blending of vegetable oils (e.g., flaxseed), as indicated on product labeling [55]. In contrast, fish and krill oils contained negligible ALA (<1% of total FA), underscoring the nutritional distinction between algal-derived and animal-derived supplements. The spider web graph further illustrates this divergence, with algal oils clustering at high ALA while fish and krill oils remain close to the origin (Figure 1).

Docosapentaenoic acids were generally scarce, but showed unique biosynthetic fingerprints. *Schizochytrium* sp. uniquely accumulated exceptionally high levels of 22:5 n-6 (42.3 ± 1.6 mg g^−1^), a recognized intermediate in the polyketide synthase pathway of DHA biosynthesis [56]. Other sources contained little to no 22:5n-6, indicating that it serves as a signature of a specific FA synthetic pathway. By contrast, 22:5 n-3 was present at low content in fish oils (1.4–28 mg g^−1^) and algal oils (3.7–8.77 mg g^−1^), while microalgae generally lacked this FA, except for *P. tricornutum*, which contained trace levels (~0.1 mg g^−1^).

Arachidonic acid (ARA, 20:4 n-6) provided another notable example of species-specific enrichment. *P. purpureum* contained very high ARA levels (27.75 ± 2.4 mg g^−1^), in agreement with recent findings [57]. Other microalgal taxa, such as *P. tricornutum* and *Nannochloropsis* sp., contained only minor amounts (<5% of total FAs). Fish oils contained moderate levels of ARA (17.76 ± 1.4 to 26.18 ± 0.83 mg g^−1^), likely reflecting dietary inputs, whereas krill oils and *Schizochytrium*-derived algal oils contained only minimal amounts. Fish tissues exhibited relatively low ARA contents (1.18 ± 0.07 to 7.6 ± 0.53 mg g^−1^), reflecting interspecies differences and feeding regimes.

The overall distribution of saturated (SFA), MUFAs, and PUFAs revealed marked variations across and within the source categories. The microalgal samples exhibited the greatest diversity in FA composition, reflecting distinct species-specific metabolic profiles. The SFA content in algae ranged from 31.26 ± 1.80% to 44.76 ± 4.03% FAs, corresponding to absolute values of 20.09–64.50 mg g^−1^. Notably, *Nannochloropsis* sp. (1) had the highest SFA content (64.50 ± 3.98 mg g^−1^), accompanied by elevated MUFA levels (28.45 ± 1.40%). In contrast, *P. purpureum*, known for its high PUFA yield, exhibited the lower MUFA proportion (15.97 ± 2.36%) and the high PUFA content (56.90 ± 1.56%), driven primarily by elevated levels of ARA and EPA. Across all algal samples, PUFA content ranged from 28.77 ± 2.73% to 59.60 ± 1.56%, while MUFAs ranged from 6.62 ± 1.92% to 31.79 ± 1.43%, highlighting the taxonomic and functional diversity of algal lipidomes.

Fish tissue samples showed greater interspecies variability, likely reflecting both physiological and dietary influences. The SFA content ranged from 33.14 ± 2.04% to 38.38 ± 1.96%, MUFA levels ranged from 13.43 ± 0.58% to 28.73 ± 1.11%, whereas PUFA proportions spanned 31.70 ± 1.12% to 53.43 ± 2.58% of the total FAs, in accordance with previous findings [58,59].

Fish oils presented relatively consistent FA profiles that aligned with the results of Mason and Sherratt (2017) [60]. On average, they contained 39.75 ± 2.07% SFA, 20.43 ± 0.89% MUFA, and 38.16 ± 1.34% PUFA, respectively. This profile reflects a typical marine lipid pattern, characterized by elevated n-3 content, reinforcing the nutritional relevance of fish oil supplements. Krill oils displayed broadly similar lipid class profiles; however, one sample contained a higher proportion of SFA (50.42 ± 1.06%). Such variation may arise from biological factors, including seasonal changes in krill diet or differences in species composition. In addition, the type of extraction process can substantially influence the lipid composition, including solvent-based extraction (e.g., ethanol, acetone, isopropanol) or enzymatic hydrolysis followed by solvent extraction [61]. Algal oils derived from *Schizochytrium* spp. were notable for their lower SFA content (~17%) and exceptionally high PUFA proportions (67–70%), highlighting their suitability as sustainable and vegetarian-friendly sources of n-3 LC-PUFAs, particularly DHA [62].

Merging absolute and relative analyses underscores that, beyond EPA and DHA, palmitoleic acid, ARA, and DPA serve as strong taxonomic markers, while ALA and LA highlight dietary and processing influences in supplements and aquaculture fish. At the same time, the overall balance of SFA, MUFA, and PUFA clearly differentiates algae, fish tissues, and oils, reinforcing both their nutritional roles and their taxonomic specificity. Together, these results provide a robust framework for distinguishing taxa and evaluating the nutritional relevance of diverse lipid sources.

### 2.2. Nutritional and Health Quality Indices

**Figure 2:**
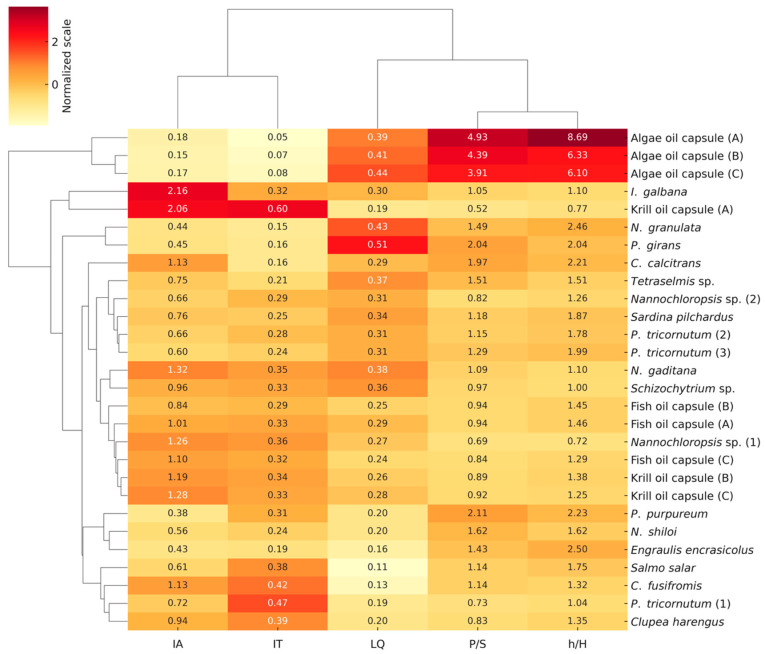
Clustered heatmap of health-related indices—index of atherogenicity (IA), index of thrombogenicity (IT), lipid quality (LQ), polyunsaturated fatty acid to saturated fatty acid ratio (P/S), and the hypocholesterolemic/hypercholesterolemic (h/H) index—calculated from fatty acid content. The heatmap displays original values with color intensities based on a normalized scale.

To enable a consistent interpretation of the health-related indices presented in Figure 2, we first outline the threshold values commonly used in nutritional lipid research to differentiate favorable from unfavorable profiles. A PUFA to SFA (P/S) ratio above 0.45 is generally associated with reduced cardiovascular and metabolic risk [63]. Lipid quality (LQ) values above 0.30 reflect EPA/DHA-rich lipid compositions of higher nutritional quality [64]. Index of atherogenicity (IA) and index of thrombogenicity (IT) values below 0.5 and 1, respectively, indicate low atherogenic and thrombogenic potential [65]. Finally, hypocholesterolemic/hypercholesterolemic FA ratio (h/H) values above 1.0, and particularly >2.0, are considered beneficial for cardiovascular health due to their association with favorable serum cholesterol modulation [66]. These threshold values serve as the basis for evaluating the relative nutritional quality of the sources analyzed.

The P/S ratio varied significantly among the sample groups, reflecting distinct profiles. Algal oil capsules displayed the highest P/S values, ranging from 3.91 ± 0.18 to 4.93 ± 0.31, reflecting a high PUFA-rich lipid composition. These P/S ratios surpassed those of all the other categories. Among microalgae, P/S ratios varied markedly. *Nannochloropsis* sp. (1) exhibited the lowest value (0.69 ± 0.02), whereas *P. purpureum* reached the highest value (2.11 ± 0.19). The latter, as well as P. *gyrans* and *P. tricornutum,* showed ratios above 1.5, suggesting a favorable degree of unsaturation for nutritional applications. In contrast, fish oil capsules showed more modest P/S ratios, ranging from 0.84 ± 0.06 to 0.94 ± 0.04. Krill oil products exhibited greater variability, with values spanning from 0.52 ± 0.03 to 0.92 ± 0.05. Among fish species, oily fish such as anchovies and sardines demonstrated intermediate P/S ratios while *Schizochytrium* sp. had a P/S ratio of 0.97 ± 0.05. Given that P/S ratios above 0.45 [63] are associated with reduced cardiovascular risk and improved lipid profiles, nearly all microalgal samples and algal oil supplements significantly exceeded this threshold. Some fish and krill oil products showed P/S ratios between 0.45 and 1.0. While these values are still within a favorable range, they represent sub-optimal P/S profiles compared to the sources studied by Chen and Liu [64].

Regarding LQ algal oil capsules performed best, ranging from 0.39 ± 0.02 to 0.44 ± 0.02. *Schizochytrium* sp. followed closely with 0.36 ± 0.02, while enriched microalgal samples derived from *Pavlova* and *Nannochloropsis* achieved even higher values of up to 0.51 ± 0.03 (Figure 2). These levels exceed those found in traditional marine sources. Fish oil capsules provided an LQ value of 0.24 ± 0.01 to 0.29 ± 0.01, with sardines showing the highest value (0.34 ± 0.01). Anchovy, salmon, and herring exhibited intermediate levels that fell within the 0.11–0.20 range, whereas krill oils ranged from 0.19 ± 0.01 to 0.28 ± 0.02, depending on the formulation and source.

The IA, which reflects the proportion of cholesterol-raising saturated FAs, varied substantially among the sources. Algal oil capsules exhibited the most favorable profiles, with IA values ranging from 0.15 ± 0.01 to 0.18 ± 0.02. Selected microalgae, including *P. purpureum* and *P. gyrans*, also performed well, with IA values between 0.38 ± 0.02 and 0.72 ± 0.07. The group mean for microalgae was approximately 0.85 ± 0.34, with several species falling well below the atherogenic threshold of 1.0 [67]. Fish oil capsules displayed moderately higher IA values (0.84 ± 0.07 to 1.10 ± 0.09), and oily fish showed species-specific variations. Anchovy was the most favorable (0.43 ± 0.02), while the herring approached 1.0 (0.94 ± 0.08). Krill oil exhibited the highest IA values overall, with one formulation reaching 2.06 ± 0.14, indicative of its relatively high saturated-fat content. *Schizochytrium* sp. displayed an intermediate IA (0.96 ± 0.08), which was less optimal than that of microalgae or its refined “algae” oil. The IT, which reflects the balance between pro-thrombogenic (clot-promoting) and anti-thrombogenic (clot-preventing) FAs in a sample, mirrored the findings in IA (Figure 2). Algal oils had the lowest IT values (0.05 ± 0.01–0.08 ± 0.01), followed by microalgae (0.15 ± 0.02–0.47 ± 0.04). The fish oils and fish tissues ranged from 0.29 ± 0.03 to 0.39 ± 0.05, while the least favorable krill oil sample reached 0.60 ± 0.05.

The h/H index, which reflects the balance between LDL-lowering and LDL-raising FAs, varied considerably among the resources. Algal oil capsules exhibited the highest and most favorable values, ranging from 6.10 ± 0.28 to 8.69 ± 0.37, indicating an exceptionally cardioprotective lipid profile (Figure 2) [68]. These values far exceed the acceptable threshold of 1.0 [66] considered to be strongly beneficial for cardiovascular health. Most microalgae, except for *Nannochloropsis* sp. (1), fell within a moderate-to-high range, from 1.04 ± 0.09 to 2.46 ± 0.25, with a group mean of approximately 1.62 ± 0.54. This suggests a generally favorable FA balance, especially in species such as *Pavlova* and *Porphyridium*, which approached or surpassed the optimal threshold. Fish oil capsules showed more modest h/H values, ranging from 1.29 ± 0.07 to 1.46 ± 0.08, indicating an acceptable but less optimal cholesterol-lowering potential. Among the whole fish, anchovy (2.50 ± 0.23) had a highly favorable h/H index, whereas herring (1.35 ± 0.12) remained in the moderate range. Krill oil showed the least favorable results, with one formulation as low as 0.77 ± 0.08, falling below the preferred 1.0 threshold value, which signals a less desirable lipid profile with limited hypocholesterolemic benefits. *Schizochytrium* sp. presented a neutral h/H value of 1.00 ± 0.09, suggesting a balanced but not particularly advantageous FA composition.

To integrate these metrics into an overall evaluation, hierarchical cluster analysis was conducted (Figure 2). This revealed a clear separation of sources: algal oils formed a distinct cluster, characterized by high P/S and LQ values, exceptionally favorable h/H indices (>6), and minimal IA and IT scores (IA < 0.2; IT < 0.1). Fish oils and whole fish grouped together, reflecting intermediate lipid health indices (IA ≈ 1.0; h/H ~1.3–2.0), while krill oils displayed heterogeneity, with two formulations clustering near fish and one (Krill oil A) separated due to its elevated IA/IT and low h/H indexes. Microalgae also showed more diverse clustering: high-performing species such as *Pavlova*, *Porphyridium*, and *Nannochloropsis granulata* aligned with the favorable groups, whereas others (e.g., *N. gaditana*, *I. galbana*) associated with more moderate profiles.

Based on these threshold values, algal oil capsules consistently satisfied all favorable criteria, combining high P/S and LQ values with exceptionally low IA and IT scores and strongly cardioprotective h/H ratios. Several microalgal species—including *P. girans*, *P. purpureum*, and *N. granulata*—also met multiple thresholds simultaneously and frequently outperformed fish sources in overall LQ. Among whole-fish samples, anchovy and sardine displayed the most favorable profiles, whereas other fish species and fish oil capsules fell primarily within moderate ranges. Krill oils, particularly Krill oil A, showed the least favorable results and failed multiple threshold criteria. Overall, this threshold-based assessment identifies algal oils and selected microalgae as the most suitable lipid sources for consumption from a cardiovascular health standpoint, while several fish and especially krill oils present comparatively less desirable profiles.

### 2.3. Glycerolipid Class Composition

**Figure 3:**
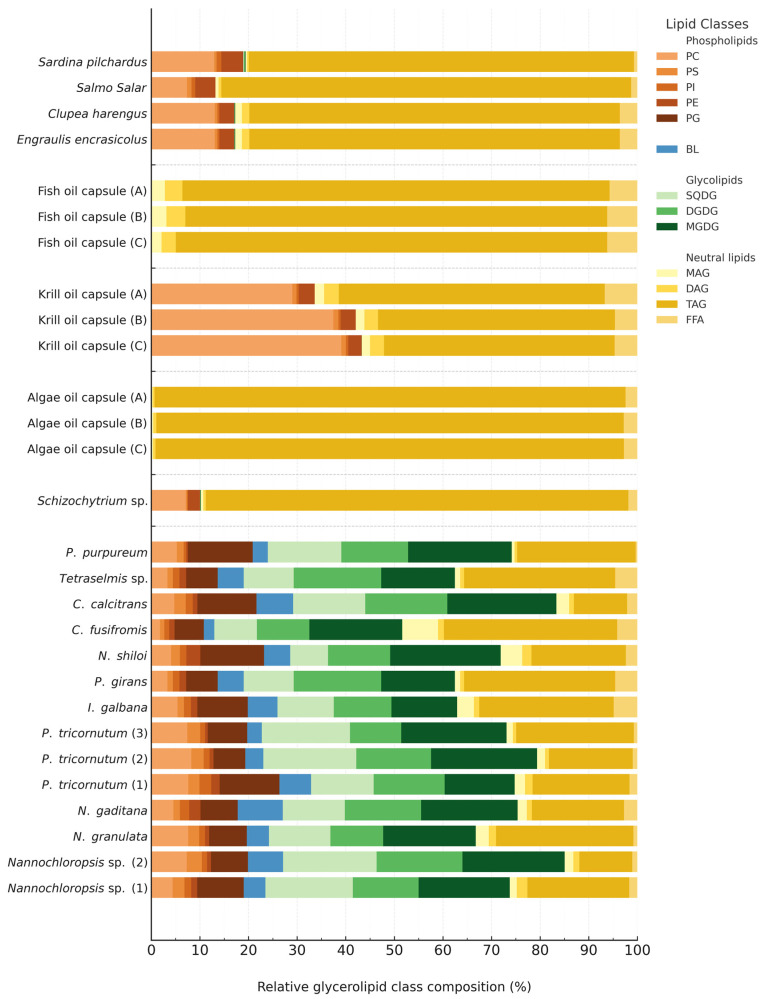
Distribution of individual glycerolipid classes—phosphatidylcholine (PC), phosphatidylserine (PS), phosphatidylinositol (PI), phosphatidylethanolamine (PE), phosphatidylglycerol (PG), betaine lipid (BL), sulfoquinovosyldiacylglycerol (SQDG), digalactosyldiacylglycerol (DGDG), monogalactosyldiacylglycerol (MGDG), monoacylglycerol (MAG), diacylglycerol (DAG), triacylglycerol (TAG), and free fatty acids (FFA)—as % of overall glycerolipid content of the studied omega-3 sources.

Following conventional GC-MS-based FA profiling, a comprehensive glycerolipid class analysis was performed using HPTLC densitometry. This approach enabled the mass-based quantification of 13 distinct lipid classes, allowing for the absolute measurement of individual glycerolipid subclasses, an underexplored aspect of the lipidome in n-3 LC-PUFA-related studies. The individual lipid class contents on a DW-basis are presented in the Supplementary Table S3, while their relative distribution in the glycerolipidome is illustrated in Figure 3.

The lipid profiles of the microalgal samples were dominated by medium-polar, chloroplast-specific glycolipids. MGDG and DGDG were among the most abundant lipid classes, with contents ranging from 15.63 ± 1.07 to 38.30 ± 1.09 mg g^−1^ and 12.07 ± 0.20 to 28.94 ± 0.91 mg g^−1^ of the DW, respectively. These accounted for approximately 14.41 ± 0.52% to 22.72 ± 1.22% (MGDG) and 10.53 ± 0.72% to 18.01 ± 0.70% (DGDG) of the total quantified glycerolipids. Other prominent lipid classes included SQDG (7.61 ± 0.31 to 35.74 ± 0.46 mg g^−1^) and PG (6.73 ± 0.28 to 19.01 ± 0.43 mg g^−1^). Collectively, these chloroplast-derived glycolipids and PG accounted for more than half of the total lipid content, except in *C. fusiformis*, underscoring their fundamental role as constituents of photosynthetic membrane structures. In contrast, the principal neutral storage lipids, TAGs, constituted a smaller proportion, ranging from 11.98 ± 0.55 to 41.66 ± 0.16 mg g^−1^, contributing 10.88 ± 0.45% to 36.77 ± 2.95% of the overall glycerolipids. Moderate quantities of phospholipids, including PC (2.01 ± 0.14 to 14.48 ± 0.60 mg g^−1^), PE (0.43 ± 0.05 to 2.90 ± 0.12 mg g^−1^), and BLs (2.39 ± 0.13 to 11.86 ± 0.45 mg g^−1^) were also observed. FFAs were detected only in minor amounts (0.47 ± 0.06 to 5.88 ± 0.45 mg g^−1^).

The predominance of membrane-forming lipids over energy storage lipids, such as TAG, aligns with the expectations for actively growing microalgae, where lipid synthesis primarily supports chloroplast and cellular membrane biogenesis. This lipid pattern is characteristic of photoautotrophic metabolism under nutrient-replete conditions. These results also corroborate prior findings that MGDG, DGDG, SQDG, and PG constitute the structural backbone of thylakoid membranes, whereas significant TAG accumulation typically occurs under nutrient-deficient conditions, such as nitrogen deprivation [20,69]. Importantly, microalgae exhibited pronounced species-specific differences in lipid class composition, which were greater than the variation observed across fish or supplement oils. For example, within *Nannochloropsis* species, TAG content differed significantly (*p* < 0.01), ranging from 17.84 ± 0.74 mg g^−1^ in *N. granulata* to 41.66 ± 0.16 mg g^−1^ in *Nannochloropsis* sp. 1. This highlights the remarkable plasticity of the microalgal lipidome and its ability to undergo dynamic metabolic remodeling in response to environmental conditions. This intrinsic variability presents an opportunity for optimization using targeted cultivation strategies. For instance, culture conditions can be manipulated to enhance glycolipid or TAG production, depending on the desired application. Notably, an increase in glycolipid content may add functional value. Recent studies suggest that n-3 FAs esterified to glycolipids exhibit bioavailability comparable to krill oil, positioning glycolipid-rich microalgae as a promising and underutilized source of dietary n-3s [70].

In contrast to the photosynthetic microalgae analyzed, the heterotrophic thraustochytrid *Schizochytrium* sp. exhibited an exceptionally lipid-rich profile dominated by neutral storage lipids. *Schizochytrium* showed the highest total glycerolipid content among all biomass-derived samples (489.73 ± 13.04 mg g^−1^), with TAG contributing 425.82 ± 12.9 mg g^−1^, or 86.95 ± 3.12% of the total glycerolipid pool (Figure 3). In contrast, chloroplast-associated glycolipids were virtually absent, and MGDG, DGDG, and SQDG were detected only in trace amounts (0.41 ± 0.03, 0.20 ± 0.01, and 0.13 ± 0.01 mg g^−1^, respectively). Their presence in *Schizochytrium* sp. likely reflects vestigial remnants of ancestral photosynthetic capabilities [71,72]. The polar lipid fraction of *Schizochytrium* primarily consisted of phospholipids. PC and PE were the most abundant, at 34.66 ± 1.83 mg g^−1^ (7.08 ± 0.37%) and 12.07 ± 0.42 mg g^−1^ (2.46 ± 0.19%), respectively. Minor amounts of PS and PI (<1.5 mg g^−1^ each) were detected, along with low contents of MAG, DAG (~2.5 mg g^−1^ each) and FFAs (9.06 ± 0.36 mg g^−1^).

Taken together, aside from a modest phospholipid fraction, *Schizochytrium*’s lipidome was overwhelmingly dominated by neutral lipids (Table 1). This composition is characteristic of *Schizochytrium* and related thraustochytrids, which can accumulate 36–84% of their dry biomass as lipids, with some strains averaging approximately 60% of their DW [62]. TAGs typically comprise 70–90% of total lipids in *Schizochytrium*, with our observed value (86.95 ± 5.15%) falling well within this reported range. Overall, *Schizochytrium* exhibits a lipid profile resembling that of oilseeds or oleaginous yeasts, characterized by large TAG reserves and limited structural lipids. This is distinct from the thylakoid membrane-dominated lipidome of photosynthetic microalgae [17,72].

As expected for high-fat pelagic fishes, TAGs were the predominant lipid class in all analyzed fish muscle samples. TAG content ranged from 88.89 ± 2.97 mg g^−1^ in sardine to 151.30 ± 9.72 mg g^−1^ in salmon. Based on the total glycerolipid content, TAGs accounted for approximately 76.21 ± 3.14% to 84.34 ± 5.42% of the glycerolipid pool, reflecting the substantial energy reserves typical for fish. The detection of trace levels of MGDG, DGDG, and SQDG in fish samples is most plausibly attributable to the presence of microalgal material. It is either introduced through dietary intake or remaining as residual gut or skin-associated content, as these lipid classes are characteristic of photosynthetic organisms. In addition phospholipids were present in appreciable amounts, indicating a contribution from the muscle cell membranes (Figure 3). PC was the most abundant polar lipid, ranging from 13.16 ± 0.62 to 25.25 ± 1.10 mg g^−1^ (7.34 ± 0.35% to 13.8 ± 0.51% of total glycerolipids), followed by PE (4.93 ± 0.33 to 7.44 ± 0.34 mg g^−1^). Together, PC and PE constituted approximately 15% of the glycerolipids, consistent with their roles as structural components of cellular membranes. Minor phospholipids, such as PI and PS, were present at lower concentrations, collectively accounting for less than 1% of the glycerolipid pool (Figure 3).

These findings confirm that the four oily fish species analyzed (sardines, salmon, herring and anchovy) maintain high TAG reserves in their muscles, contributing not only to metabolic energy storage but also potentially to buoyancy regulation [73]. Nonetheless, a consistent baseline of phospholipids (~15% of total glycerolipids) was observed, reflecting the structural requirements of muscle cell membranes. These findings are consistent with expectations, as fish body oils are stored primarily as TAG in adipose tissue [74]. The observed PC:PE ratio of approximately 3:1 aligns with the established profiles of animal cell membranes, where PC predominates [75]. This ratio may influence membrane fluidity and lipid–protein interactions in muscle tissue. Moreover, EPA and DHA are reported to proportionally enrich polar membrane lipids, where they can comprise up to ~50%, consistent with the physiological principle that only surplus n-3 LC-PUFAs are deposited into storage lipids [76]. The substantial TAG abundance observed in our fish samples is therefore indicative of such a surplus state and accordingly may predict that the total EPA and DHA pool will predominantly reside in TAG—reaching up to 80–90%. This distinction has nutritional implications, as lipid classes influence both the bioavailability and metabolic fate of n-3 LC-PUFAs.

The lipid class composition of commercial n-3 capsules varied considerably depending on the oil source used. Fish oil capsules, derived from fish body oils, consisted almost entirely of neutral lipids (Figure 3, Table 1). On average, these oils contained 756.52 ± 10.80 to 817.94 ± 23.46 mg g^−1^ of TAG, representing 86.8 ± 2.6% to 88.76 ± 3.76% of the total glycerolipids. Minor components included MAG (2.1 ± 0.15% to 3.12 ± 0.09%), DAG (2.94 ± 0.22% to 3.89 ± 0.13%), and FFAs (5.71 ± 0.18% to 6.2 ± 0.36%). Notably, no phospholipids were detected in the fish oil capsules. This is consistent with the use of adipose-derived raw material—naturally low in membrane phospholipids—and with standard refining steps such as degumming and neutralization, which deliberately remove residual polar lipids. Consequently, commercial fish oils are characteristically TAG-enriched and largely depleted of phospholipids [74]. However, the presence of partial glycerides and FFAs suggests incomplete or unstable re-esterification during processing. This residual ~10 to 12% non-TAG content likely results from partial hydrolysis, oxidative degradation, or inefficiencies in enzymatic or chemical re-esterification. While such compositions are typical of commercial-grade oils, they may influence the oxidative stability and digestibility. These findings underscore the distinct lipidomic signature of refined fish oils compared to whole-organism sources, in which membrane phospholipids are preserved.

In contrast, krill oil capsules exhibited a markedly different lipid class profile, characterized by a substantial phospholipid fraction (Figure 3). PC was the dominant polar lipid, present at 228.7 ± 10.91 to 330.45 ± 18.14 mg g^−1^ (29.0 ± 1.38% to 39.08 ± 2.15%), accompanied by smaller amounts of PE (23.33 ± 1.52 to 26.73 ± 1.70 mg g^−1^; 2.76 ± 0.18% to 3.28 ± 0.15%). PS and PI contributed less than 1% combined. Overall, phospholipids comprised approximately one-third of the overall glycerolipids in krill oil. Consistent with this, TAG content was significantly lower (401.23 ± 22.59 to 431.21 ± 24.02 mg g^−1^; 47.45 ± 2.67% to 54.69 ± 3.05%). MAG, DAG and FFAs accounted for an additional 10 to 12% of the total glycerolipidome. This composition aligns with established profiles for krill oil, which typically contains 30 to 40% phospholipids, predominantly PC, while the remainder is composed mainly of TAG. The enrichment of phospholipids in krill oil is nutritionally important. Unlike fish oil, krill oil contains a substantially higher proportion of PC (~30%), which contributes to membrane fluidity, lipid transport, and cellular signaling [77]. Owing to this elevated PC content, n-3 FAs in krill oil are largely phospholipid-bound and are reported to have higher bioavailability than TAG-bound forms, thereby enhancing the functional efficacy of krill oil as a lipid delivery system [21].

Algal oil capsules derived from *Schizochytrium* (or similar heterotrophic microalgae) exhibited a lipid class composition closely matching that of fish oil (Figure 3). In our formulation, the DHA-rich oil contained 764.23 ± 4.05 to 850.70 ± 43.00 mg g^−1^ TAG, representing ~96% of total lipids. Phospholipids (e.g., PC, PE) were undetectable, while minor fractions included FFAs (≈2–3%) and mono-/diacylglycerols (<0.65% each). These data indicate that industrial extraction and refining yielded a nearly pure TAG-based DHA oil.

Among commercial n-3 supplements, the main distinction lies between TAG-dominant oils and phospholipid-rich formulations. Fish body oils and *Schizochytrium*-derived algal oils typically contain >75 to 85% TAG, negligible phospholipids, and traces of partial glycerides. In contrast, krill oil contains substantially less TAG (~40%) and a significant phospholipid fraction (~30–40%), primarily PC [77,78,79]. This structural enrichment has nutritional implications, since EPA and DHA bound to phospholipids have been shown to exhibit enhanced bioavailability, improved membrane incorporation, and supply of choline, a key nutrient for lipid transport and cell signaling [51,61,80]. Fish muscle lipids further illustrate these distinctions, with ~75 to 85% TAG and ~10 to 20% phospholipids (mostly PC and PE), reflecting dual functions in energy storage and membrane maintenance. Refining steps, however, largely eliminate these polar lipids, yielding oils dominated by storage TAGs. Additionally, storage and processing can increase mono- and diacylglycerol content due to hydrolytic reactions [77,81,82].

Taken together, the glycerolipid class profiles show that the examined n-3 sources differ in both the types and proportions of structural and storage lipids they provide. Commercial fish and algae oils were dominated by TAG, with glycolipids and phospholipids absent, whereas fish muscle retained a modest phospholipid fraction consistent with the membrane-derived origin of the tissue. Krill oil, however, retained a markedly higher phospholipid content—primarily PC—which differentiates it structurally from both fish and algal oils. Photosynthetic microalgae further contributed source-specific polar lipids, including PG and characteristic glycolipids. Phospholipids have attracted increasing interest in nutritional and lipidomic research due to their established roles in membrane organization, emulsification, and lipid transport, and their emerging use as structural matrices for formulating bioactive lipids [83,84]. The present dataset shows that only a subset of n-3 sources—most prominently krill oil and, to a species-dependent extent, microalgae—naturally provide substantial phospholipid fractions, whereas refined oils supply n-3 lipids almost exclusively in TAG form. These differences in phospholipid availability therefore represent a meaningful comparative dimension of our study, distinguishing structurally phospholipid-rich from phospholipid-poor resources and providing a basis for interpreting their potential applications in nutrition, formulation, and functional lipid delivery.

### 2.4. Quantitative EPA and DHA Distribution by Glycerolipid Class

Subsequent to the initial quantification of lipid classes via HPTLC densitometric analysis, the integrated HPTLC–GC–MS platform was employed for a more detailed compositional characterization. This approach enabled the identification and absolute quantification of EPA and DHA in each glycerolipid class.

**Figure 4:**
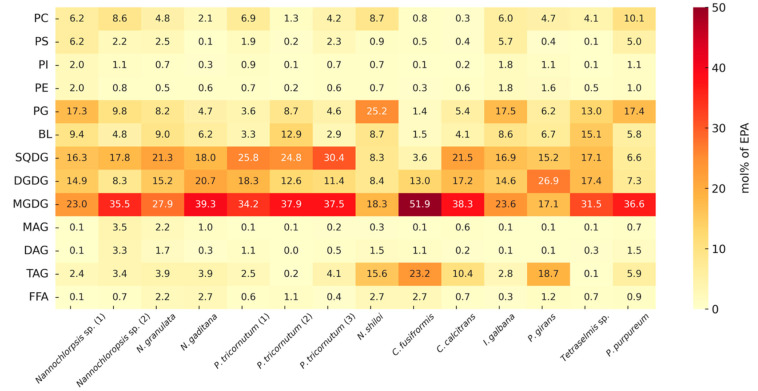
Heatmap of eicosapentaenoic acid (EPA) distribution in glycerolipid classes in selected microalgal species. The values represent average mole percentages (mol %). Each column represents the mol % contribution of the overall EPA content found in the given glycerolipid class.

**Figure 5:**
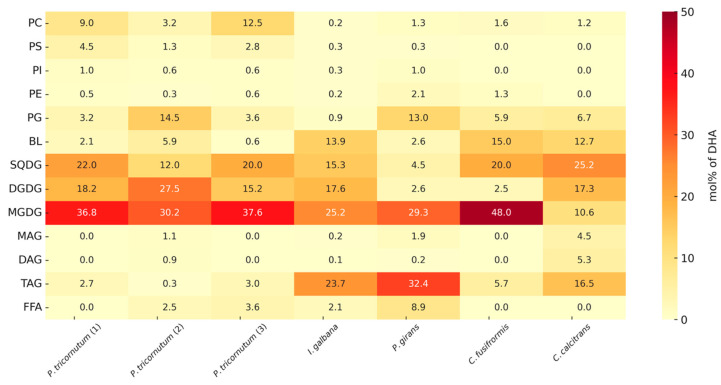
Heatmap of the docosahexaenoic acid (DHA) distribution in glycerolipid classes in selected microalgae species. The values represent average mole percentages (mol %). Each column represents the mol % contribution of the overall DHA content found in the given glycerolipid class.

The distribution patterns of EPA and DHA across the different glycerolipid classes exhibited a marked degree of uniformity in most of the microalgal species analyzed. As illustrated in Figure 4 and Figure 5 these n-3 LC-PUFAs were predominantly associated with medium-polar thylakoid membrane lipids, particularly the glycolipids MGDG, DGDG, SQDG, and the phospholipid PG. In contrast, neutral storage lipids, such as TAG, along with most other phospholipids, contained only minor proportions of these FAs. In fact, in every species analyzed, over 50% of the total EPA and DHA content was localized within MGDG, DGDG, SQDG, and PG. In many cases, this proportion was substantially higher, often ranging between 70 and 80%. Conversely, TAG and other neutral lipids contributed only a small fraction to the overall EPA and DHA pool. This distinct partitioning highlights the critical role of lipid classes in modulating PUFA allocation within the microalgal lipidome [85].

Quantitatively, the chloroplast glycolipids together accounted for the majority of EPA and DHA content. Combined, MGDG, DGDG, and SQDG accounted for ~60% of EPA on average or ~73% when PG is included. MGDG was the single largest reservoir of EPA, containing 30.8 ± 10.8% of the total EPA across the microalgal samples (Figure 4). This dominance of MGDG is consistent with its role as an endpoint lipid class for EPA in chloroplast membranes [86]. The other chloroplast glycolipids also contained substantial shares: SQDG and DGDG contained approximately 17.0 ± 7.4% and 14.0 ± 5.8% of total EPA, respectively (and very similar fractions of DHA, ~17.0% and ~14.4%); PG, a crucial phospholipid in photosynthetic membranes, contributed an additional 11.0 ± 7.3% of EPA (averaged across species) and approximately 6.8 ± 5.1% of DHA. Thus, in most algae the chloroplast lipids (MGDG, DGDG, SQDG, and PG) together dominated the EPA and DHA distribution, often sequestering well over two-thirds of these FAs, even with species differences. Phospholipid classes located outside the chloroplast (PC, PE, PI, and PS) served only as minor reservoirs of EPA and DHA (Figure 4 and Figure 5). PC typically accounted for a single-digit percentage of total EPA (~4–5% on average), while PI contributed less than 1%. Even when summed together, all non-PG phospholipids generally accounted for less than 10% of the EPA or DHA in a given sample. Betaine lipids (e.g., diacylglyceryl trimethylhomoserine), which are phosphate-free polar lipids found in many algae, consistently contained a moderate share of these PUFAs (~7% of total EPA or DHA on average). Notably, the presence of ~5–15% EPA in BL supports the hypothesis that betaine lipids can act as transient reservoirs or intermediates in the intracellular redistribution of EPA, potentially channeling it toward galactolipids such as MGDG and DGDG [87]. This interpretation is further reinforced by recent evidence that betaine lipids may function as acyl group donors during membrane remodeling [88]. Overall, our observations for polar and medium-polar glycerolipids are consistent with previous reports [24,89]. In contrast to polar lipids, neutral lipids contained only minor fractions of EPA and DHA. TAG averaged ~6.7% of total EPA, and, in the majority of the species, it was less than 5% of the EPA [90,91]. FFAs, MAG and DAG were each also negligible contributors (generally in the range of 1–3%). Even under conditions promoting TAG accumulation, microalgae like *Nannochloropsis* channel very little EPA into TAG [91].

The DHA distribution mirrored that of EPA in most respects, reinforcing the general trend that chloroplast lipids dominate PUFA allocation. As shown in Figure 5, MGDG was the principal DHA-bearing lipid (approximately 30% on average), with SQDG and DGDG providing another ~30% combined. In total, the glycolipids plus PG encompassed ~69% of DHA on average (and at least ~50% of DHA in every assessed species). This reflects a shared biochemical partitioning: like EPA, DHA in microalgae predominantly accumulates in polar lipids rather than in neutral storage oils [92]. A subtle difference for DHA was a slightly greater relative presence in TAG in certain DHA-rich species. For instance, the haptophytes *I. galbana* and *P. gyrans* showed elevated DHA in TAG (approximately 20–30% of total DHA), which raised the mean TAG contribution of DHA to ~12%. Nevertheless, even in these outliers, the majority of DHA remained associated with glycolipids and other polar lipids. In contrast, the phospholipids PC and PE played only a minor role in DHA distribution (typically <5%). Overall, the data demonstrate that whether an n-3 is EPA or DHA, the cell largely partitions it into chloroplast-associated lipids (glycolipids and PG) that fulfill structural and functional roles in membranes [20]. The scarcity of EPA and DHA in TAG and other neutral lipids suggests that these FAs are not substantially stored in lipid droplets but are rather retained in membrane lipids, where they contribute to essential cellular functions [90,91]. This pattern, high EPA/DHA in glycolipids and PG, low in TAG, is consistently observed across diverse photosynthetic microalgae and underscores the importance of lipid class composition in determining the fate and bioavailability of n-3 FAs in algal cells [69,92]. An important exception is found in thraustochytrids such as *Schizochytrium*, which do not rely on the membrane-linked desaturase/elongase pathway but instead synthesize DHA predominantly via a large polyketide synthase (PKS) complex located in the cytoplasm [62]. Because the PKS route is decoupled from membrane lipid metabolism, the newly produced DHA is rapidly channeled into the acyl-CoA pool and efficiently incorporated into TAG stored in cytoplasmic oil bodies. As a result, *Schizochytrium* accumulates DHA almost exclusively in neutral lipids, yielding oil compositions that closely resemble fish oil supplements as nearly pure TAG-based DHA carriers [56,93].

In all the other sources, the EPA and DHA distributions demonstrated a more distinct pattern, and thus were not represented on the heatmaps. The EPA and DHA distribution in the oily fish samples, as expected from the glycerolipid composition analysis, displayed a TAG-dominated profile. The same was observed for the purified fish oil capsules. Across the fish species analyzed, over 90% of EPA and DHA were found in TAG, with the remaining divided among minor lipid classes, such as PC, PE and FFA. In sardine, TAG held ~94% of the EPA and ~91% of the DHA. In salmon, TAG contained ~96% of EPA and ~93% of DHA. In herring, the TAG content was slightly lower but still dominant and contained ~89% of EPA and ~85% of DHA. The residual EPA and DHA were mostly found in PC (~3–5%), PE (~1–3%), and FFA (~2–4%), likely originating from cell membrane remnants or minor lipid hydrolysis during lipid extraction. This pattern aligns with the known lipid physiology in fish, where EPA and DHA are predominantly stored in triacylglycerols in the tissues of oily species. In lean fish such as cod, however, EPA and DHA are primarily associated with membrane phospholipids rather than TAG, highlighting an important distinction in lipid distribution across different fish categories. The phospholipid fraction, while essential for membrane function, represents a smaller portion of the total lipid content in whole-body oils derived from most fish tissues [94,95].

In fish oil capsules, typically, over 85 to 95% of the EPA and DHA resided in TAG, with the remainder mostly accounted for by minor lipid breakdown products, such as FFA and partial glycerides (MAG, DAG). For instance, one fish oil capsule sample had approximately 90% DHA in TAG, approximately 5 to 8% as FFA and a few percent as MAG/DAG, reflecting slight hydrolysis during processing or storage. The algal oil capsules exhibited an EPA/DHA distribution similar to that of fish oil. In all three algal oil supplements analyzed, over 95 to 98% of the EPA DHA was contained in TAG, with only trace levels in the polar lipids. These supplements are typically derived from the heterotrophic protist *Schizochytrium* sp. and essentially consist of purified TAG oils. Our data confirm that algal n-3 supplements deliver FAs predominantly in the TAG form, analogous to fish oils. Minor components, such as FFA and MAG/DAG, were present at low levels (generally <3–4% combined), indicating a high degree of intact TAG.

In contrast, krill oil capsules showed a phospholipid-dominant distribution of EPA and DHA. In our krill oil samples, approximately 68–75% of the total EPA and DHA was found in the PC fraction, with additional DHA apportioned to other phospholipids, such as PE. Only approximately 17–21% of the DHA in krill oil was present in TAG, confirming that n-3s in krill are mostly carried by phospholipids. This aligns with the known compositional differences between krill and fish oils: fish store FAs predominantly as TAG in adipose tissues, whereas krill incorporate a large fraction (approximately one-third to two-thirds) of their FAs into phospholipids [80,96]. Indeed, krill oil has been reported to contain approximately 78% of EPA and 79% of DHA in the form of phospholipid-bound species, whereas only negligible amounts of these FAs are associated with TAG. These reports are in good agreement with our current findings, indicating that a substantial proportion of DHA (approximately 70%) is incorporated into the PC fraction. This lipid class-specific distribution underscores the preferential incorporation of LC-PUFAs into membrane-associated phospholipids in marine-derived lipid sources, such as krill oil.

From a nutritional and biochemical perspective, these findings are of particular importance. The form in which EPA and DHA are delivered, whether as TAG, phospholipids (e.g., PC), or glycolipids, directly influences their digestion, absorption, and metabolic fate in humans. TAG-bound n-3s, as found in fish and *Schizochytrium*-derived oils, undergo hydrolysis by pancreatic lipase, re-esterification in enterocytes, and incorporation into chylomicrons. In contrast, phospholipid-bound n-3s, such as those in krill oil, may exhibit enhanced bioefficacy due to their more efficient incorporation into cell membranes and alternative absorption routes, such as via lyso-PC (LPC) intermediates. For instance, emerging evidence suggests that phospholipid or lysophospholipid-bound DHA may facilitate greater DHA accretion in neural tissues and erythrocytes compared to TAG-bound DHA. Lipid-carrier forms such as LPC-DHA are actively transported across the blood–brain barrier by the major facilitator superfamily domain–containing protein 2a (Mfsd2a), which experimental models suggest can result in higher brain enrichment compared to TAG-DHA [97,98,99]. These findings highlight the critical importance of DHA carrier form in determining its bioavailability and tissue distribution. Given the increasing recognition of the close interplay between lipid metabolism and amyloidogenic processes, improved delivery of DHA to neuronal tissues may provide a mechanistic basis for its reported benefits in Alzheimer’s disease. Consistent with this, DHA supplementation is currently being evaluated in phase II clinical trials (NCT02578523; [100]). Thus, a more detailed investigation of the differential effects of dietary n-3 FAs on lipidation processes and their influence on Aβ pathology appears highly promising for guiding the development of nutritional adjunctive therapies targeting neurodegenerative disorders.

Glycolipid-associated EPA and DHA, found in native microalgae, represent a relatively underexplored yet potentially valuable delivery form. Although their digestibility and bioaccessibility in humans remain to be fully characterized, these lipid classes have been proposed to exert additional bioactivities, although evidence in humans is limited. Accordingly, microalgal biomass enriched in glycolipid-bound PUFAs could serve not only as a sustainable source of n-3s but also as a multifunctional nutraceutical ingredient [101,102].

These insights may suggest several possibilities for future research. Nutritional formulation strategies could be tailored to specific lipid classes, for example, PC-rich formulations for cognitive support, TAG-rich supplements for general health, or glycolipid-enriched formulations as an emerging area of investigation.

Digestibility and uptake studies will be essential for clarifying absorption kinetics and tissue distribution of glycolipid-bound EPA and DHA in humans and animal models. Advances in algal strain development may allow the optimization of culture conditions to direct PUFA allocation into the desired lipid fractions, for example, by increasing the glycolipid content for functional applications.

In conclusion, lipid class matters, not only in determining the intracellular localization and metabolic function of EPA and DHA in source organisms, but also in potentially helping to shape their nutritional quality, bioavailability, and functional potential in food, feed, and health applications. Thus, a glycerolipid class–specific approach is critical for advancing our understanding of n-3 sources and for guiding the development of more effective EPA- and DHA-rich nutritional formulations.

## 3. Materials and Methods

### 3.1. Chemicals

All analytical-grade solvents and HPTLC plates (silica gel 60 F_254_; 20 × 10 cm and 10 × 10 cm glass-backed), copper(II) sulfate heptahydrate, potassium chloride, orthophosphoric acid (85% *w*/*w*), sulfuric acid (96% *w*/*w*) and hydrochloric acid (37% *w*/*w*) were obtained from Carl Roth (Karlsruhe, Germany). Primulin dye was purchased from Merck (Darmstadt, Germany). Lipid standards were also purchased from Merck and included PC (15:0–18:1), PE (15:0–18:1), PI (18:1–18:1), PS (18:1–18:1), PG (16:0–18:1), MGDG (18:1–18:1), DGDG (18:3–18:3; main species), SQDG (18:3–16:0; main species), DGTS (16:0–16:0), EPA (as FFA), triolein (as TAG), diolein (as DAG), and monoolein (as MAG). The 37-Component FAME Mix (Supelco, purchased from Merck) was used for calibration and retention time identification of the peaks. Trans-10-heptadecenoic Acid (C17:1) was purchased from Biomol GmbH, (Hamburg, Germany) and used as an internal standard in FAME analysis after methanolic transesterification.

### 3.2. Sample Origin and Preparation

Various industrially produced known n-3 LC-PUFA-producing microalgal species were obtained. *Nannochloropsis* sp. (1), *Nannochloropsis gaditana*, *Phaeodactylum tricornutum* (1), *Chaetoceros calcitrans*, *Cylindrotheca fusiformis*, *Nanofrustulum shiloi, Pavlova gyrans*, *Isochrysis galbana, Tetraselmis* sp. were obtained as freeze-dried powders whereas *Nannochloropsis* sp. (2), *Nannochloropsis granulata, P. tricornutum* (2), *P. tricornutum* (3). *Porphyridium purpureum* and *Schizochytrium* sp. were obtained as concentrated frozen biomass. These microorganism samples were kindly provided by Necton (Olhão, Portugal), Algenfarm Klötze GmbH (Klötze, Germany), and Salata AG (Ritschenhausen, Germany). Only one batch per species or strains was available. Each biomass was analyzed as supplied, following a 5 g subsampling step to ensure sufficient and homogeneous material, and subsequently subjected to triplicate determination. Marine animal samples were sourced from a local fish market in Leipzig (Germany) and analyzed in the form typically consumed by humans. Atlantic salmon (*Salmo salar*, farmed) and Atlantic herring (*Clupea harengus*, wild) were purchased from an ice display and kept chilled whereas European anchovies (*Engraulis encrasicolus*, wild) and European sardines (*Sardina pilchardus*, wild) were obtained as frozen imported products. To obtain a representative sample for each species, tissue from several individuals was combined and homogenized and analyzed as triplicates. Commercially available n-3 supplements in the form of oil capsules were purchased online, including fish-, krill-, and algae-derived oils. Fish oil capsules A, B, and C contained 1000, 1000, and 500 mg of oil, respectively. Krill oil capsules A, B, and C contained 600, 590, and 520 mg of oil, respectively, whereas algae oil capsules A, B, and C contained 481, 500, and 1000 mg of oil, respectively, derived from *Schizochytrium*. Each capsule originated from a different manufacturer. Because the aim was to assess the representative composition of each product rather than capsule-to-capsule variability, six to eight capsules per product were randomly selected, punctured, and the extracted oils pooled and homogenized to generate a batch-level composite sample. These composite samples were subsequently analyzed in triplicate.

### 3.3. Sample Processing

The concentrated frozen biomass samples and the fish samples were freeze-dried (Christ^®^ Alpha 1–4). From all lyophilized sample 100 mg was mixed with approximately 0.5 mL (diameter: 1.0 mm) and 0.3 mL (diameter: 0.1 mm) glass beads to which 100 μL of chloroform: methanol (2:1, *v*/*v*) was pipetted and vortexed thoroughly. Biomass was disrupted for 10 min at a frequency of 30 s^−1^ by the vibration mill MM400 (Retsch, Haan, Germany). For the disintegrated biomass, 1 mL of ice-chilled chloroform: methanol (2:1, *v*/*v*) was pipetted, vortexed thoroughly, and centrifuged for 1 min at 15,100× *g*. The supernatant was transferred into a 10 mL glass tube. The extraction process was repeated nine times and performed on ice in the absence of light. To the pooled lipid extract, 2 mL of 1 M NaCl solution was added and mixed thoroughly. After phase separation, the lower organic phase was collected and were dried under reduced pressure using a hei-vap precision rotary evaporator (Heidolph, Germany) at 20 °C and stored at −85 °C in nitrogen-flushed glass containers until further analysis. The n-3 supplements were analyzed in their fresh form. The required amount of oil was obtained as described in Section 3.2. Similarly to the lyophilized samples 100 mg was used for analysis.

### 3.4. HPTLC-GC-MS

HPTLC densitometry and GC-MS analyses were conducted as previously described with minor modifications [20]. In brief: HPTLC Silica gel 60 F_254_ plates were pre-washed in a glass development chamber with isopropanol until the solvent front reached 9 cm. After drying, plates were activated on a plate heater (CAMAG, Muttenz, Switzerland) at 130 °C for 60 min. The lipid extracts and standards were applied using a Linomat 5 (CAMAG), controlled by the visionCATS 4.0. software (CAMAG). The application was performed 8 mm from the lower edge of the plate as 8 mm bands with a center-to-center spacing of 11.4 mm. A constant application rate of 150 nL s^−1^ was used under continuous drying with compressed air at 6 bar. Sample volumes between 2 and 10 µL were applied, depending on the lipid content of each extract. Prior to plate development, the plate was conditioned at a relative humidity of 33% using a saturated solution of magnesium chloride for 10 min, followed by saturation of the chamber for 20 min (with saturation pad) in the automatic Development Chamber (ADC 2) (CAMAG).

To account for differences in glycerolipid class composition among the samples, each extract was analyzed using a minimum of two HPTLC plates. One plate was developed in n-hexane/diethyl ether/glacial acetic acid (70:30:1, *v*/*v*/*v*) to a solvent front of 90 mm. The second plate was developed in a polar solvent system composed of methyl acetate/isopropanol/chloroform/methanol/0.25% aqueous KCl, acidified with glacial acetic acid (25:25:25:10:4.35, *v*/*v*/*v*/*v*/*v*), using the same 90 mm elution distance.

For quantitative detection of glycerolipid classes, developed plates were derivatized by dipping for 6 s in a modified copper sulfate reagent, prepared by dissolving 20 g CuSO_4_·7H_2_O in 200 mL methanol and acidifying the solution with 8 mL of 96% (*w*/*w*) H_2_SO_4_ and 8 mL of 85% (*w*/*w*) H_3_PO_4_. Plates were dried under a stream of cold air and heated at 140 °C for 30 min. Visualization was performed at 520 nm using a TLC Scanner 4 with visionnCATS 4.0 software (CAMAG). Glycerolipid spot intensities were integrated based on peak area without data smoothing and are expressed in arbitrary units.

For analysis of FA distributions in glycerolipid classes, lipid extract was applied on a 10 × 10 cm plate as a 65 mm broad band. The co-running lipid standards that were applied on the same plate as 8 mm bands with an 11 mm distance from the broad band of the sample and separated using the different solvent systems mentioned above. After development, lipid bands were visualized with primulin (0.05 *w*/*v* in Acetone (80% *v*/*v*) and identified, marked with a pencil, scraped off with a scalpel, and transferred to glass tubes with screw-cap lids. Lipids were extracted from the silica with 3 mL of methanol by vortexing for 1 min. Tubes were centrifuged at 4225× *g* for 5 min at 4 °C, and the supernatant was filtered through a 0.22 µm polytetrafluoroethylene (PTFE) membrane. Subsequently, 1 mL of 12 M HCl and a known amount of internal standard (C17:1) were added. Samples were trans esterified for 1 h at 95 °C in a water bath. After cooling, 0.4 mL pre-filtered n-hexane and 3 mL deionized water were added and vortexed for 30 s. Following phase separation, the n-hexane layer was transferred to GC vials for FAME analysis. FAMEs were analyzed using an Hewlet-Packard (Waldborn, Germany) 5972 GC–MS system equipped with Hewlett-Packard ChemStation software version B.03.01. Ionization was performed at 70 eV and 230 °C. The injector (spitless mode) and interface were maintained at 250 °C, and helium was used as carrier gas at a constant flow of 0.9 mL min^−1^. Separation was carried out on a BPX-70 capillary column (SGE, Melbourne, Australia; 30 m × 0.32 mm i.d., 0.25 µm film thickness). The oven program was as follows: after a 2 min hold at 50 °C, the temperature was increased to 179 °C at 8 °C min^−1^ and held for 0.05 min; then increased to 185 °C at 1 °C min^−1^ and held for 0.05 min; followed by heating at 8 °C min^−1^ to a final temperature of 250 °C. All results expressed as mg g^−1^ refer to the DW basis of each sample.

### 3.5. Calculation of Nutritional and Health Quality Indices

The nutritional and health-promoting properties of the samples were assessed using five indices derived from their FA composition. These indices include EPA and DHA contribution as LQ, P/S ratio, IA, IT and h/H. Calculations were performed using the formulas described by Chen and Liu [64].

(1) $$LQ=\frac{C20:5n-3+C22:6n-3}{\sum FA}$$

(2) $$IA=\frac{C12:0+4\times C14:0+C16:0}{\sum Unsaturated\;FA}$$

(3) $$IT=\frac{C14:0+C16:0+C18:0}{0.5\times MUFAs+0.5\times n-6PUFAs+3\times n-3PUFAs+\frac{n-3PUFAs}{n-6PUFAs}}$$

(4) $$h/H=\frac{C18:1(cis)+\sum PUFA}{C12:0+C14:0+C16:0}$$

### 3.6. Statistical Analysis

The processed data were visualized using Pandas (v2.1.3), NumPy (v1.27.5), SciPy (v1.11.3), Matplotlib (v3.8.0), and Seaborn (v0.13.2). Data were illustrated using multiple visualization methods, including stacked bar charts spider web graphs, clustered heatmaps, and heatmaps to compare lipidomic patterns across sources. Differences in lipidomic profiles were assessed using two-way ANOVA. Levene’s test was conducted to evaluate the equality of variances. If the homogeneity test yielded significant results, the Brown-Forsythe correction was applied to account for variance heterogeneity. Two-way ANOVA was followed by Tukey’s post hoc test for multiple comparisons. A *p* < 0.01 was considered statistically significant. All statistical analyses were performed using the SciPy (v1.11.3) software package.

## 4. Conclusions

This study delivers the first comprehensive glycerolipid class-resolved quantification of EPA and DHA across algal, protist, fish, and supplement sources, together with detailed lipid signatures including glycerolipid and FA contents. Using an HPTLC–GC–MS workflow across 12 microalgae species, *Schizochytrium* sp., four oily fish, and nine commercial oils, we quantified 13 lipid classes and mapped EPA and DHA into each. Photosynthetic microalgae localized the majority of EPA (and DHA when present) to chloroplast glycerolipids (MGDG, DGDG, SQDG, PG), confirming their structural role in photosynthetic membranes. By contrast, *Schizochytrium* and oily fish stored n-3 LC-PUFAs almost exclusively in triacylglycerols, reflecting their function as energy reserves. Krill oils exhibited a distinct phospholipid-rich profile, with DHA and EPA predominantly bound to PC, a carrier form associated with enhanced bioavailability.

When interpreted against commonly referenced nutritional thresholds, the derived health indices revealed favorable profiles across several source categories, each for different reasons. Algal oil capsules generally showed high P/S and h/H values and low IA and IT values, while certain fish species—such as anchovy and sardine—and fish oils also exhibited nutritionally favorable index patterns. Krill oils tended to fall within moderate but still favorable ranges for several indices. *Schizochytrium* biomass and many microalgal species showed variable index values, with some aligning with the more favorable groups and others displaying intermediate characteristics. Taken together, these results indicate that no single source is uniformly optimal across all criteria; rather, each source provides EPA and DHA within a distinct combination of lipid-class characteristics and health-related indices.

Looking ahead, and based on these findings, we aim to combine HPTLC–GC–MS with HPTLC–LC–MS to achieve absolute quantification of individual lipid molecular species and gain molecular-level insight into the distribution of EPA and DHA across glycerolipid classes. The present workflow already demonstrates high reproducibility in tracking lipid-class composition across diverse n-3 sources, which supports its use in future studies aiming to evaluate how glycolipid-, phospholipid-, and TAG-bound n-3 FAs differ in digestion and uptake. To deepen the link between lipid structure and biological relevance, coupling class-resolved lipidomics with standardized digestion models such as INFOGEST will be essential to determine how glycolipid-, phospholipid-, and TAG-bound EPA and DHA differ in bioaccessibility, uptake, and tissue targeting. In parallel, the integration of HPTLC-based effect-directed analysis (EDA) may further help associate chemical profiles with functional bioactivity in a controlled manner. Together, these planned complementary approaches will enable the correlation of lipid signatures with absorption and bioactivity profiles, contributing to a more mechanistic understanding of lipid structure–function relationships. This integrative strategy may advance understanding of the structure–uptake–function relationships of n-3 carrier forms and inform the evidence-based development of lipid carriers and nutraceutical formulations.

## Figures and Tables

**Table 1:** Comparative lipid profiling of microalgae, *Schizochytrium* sp., fish, and supplement groups. Lipid contents are expressed as mg g^−1^ dry weight (DW). Saturated fatty acids (SFA), monounsaturated fatty acids (MUFA), polyunsaturated fatty acids (PUFA), eicosapentaenoic acid (EPA), docosahexaenoic acid (DHA), recommended daily intake (RDI). n-3 and n-6 stand for omega-3 and omega-6 FAs.

	Fish Species	Fish Oil Capsules	Krill Oil Capsules	Algae Oil Capsules	*Schizochytrium* sp.	Microalgae
Σ Phospholipids [mg g^−1^]	21.19–33.02	0.00	265.04–366.00	0.00	49.15	12.06–43.52
∑ Betaine lipids [mg g ^−1^]	0.00–0.02	0.00	0.00	0.00	0.00	2.39–11.86
Σ Glycolipids [mg g^−1^]	0.00–0.53	0.00	0.00–0.14	0.00	0.74	39.66–100.01
Σ Neutral lipids [mg g^−1^]	90.43–160.13	860.64–921.49	479.42–523.46	788.46–882.87	439.84	18.22–52.21
Overall glycerolipid content [mg g^−1^]	112.17–193.52	860.64–921.49	788.49–859.47	788.46–882.88	489.72	97.87–198.92
∑ SFA [mg g^−1^]	36.05–60.50	324.25–350.02	280.39–359.67	110.04–145.01	187.27	20.09–64.50
∑ MUFA [mg g^−1^]	18.19–37.86	164.99–168.16	140.73–185.20	109.36–121.66	46.22	6.24–42.86
∑ PUFA [mg g^−1^]	40.99–86.49	295.13–311.82	187.05–257.70	528.93–566.99	181.40	32.62–68.24
C20:5 (n-3) [mg g^−1^]	9.37–14.23	142.44–176.49	91.22–120.04	112.34–119.48	18.31	8.20–51.00
C22:6 (n-3) [mg g^−1^]	9.41–25.02	40.29–94.27	44.05–78.17	178.82–259.10	142.10	0.00–14.63
EPA+DHA RDI [g] *	7.24–13.31	1.06–1.19	1.26–1.85	0.67–0.84	1.56	4.90–20.87
n-3/n-6 [-]	1.36–9.54:1	6.2–6.68:1	5.48–7.03:1	6.75–7.39:1	3.76:1	0.56–15.61:1

* Range is calculated using the minimal RDI target of 250 mg. The lower bound represents the best-case scenario, corresponding to the amount required, on a dry weight basis, from the highest EPA+DHA producer within the group. The upper bound represents the worst-case scenario, based on the amount required from the lowest EPA+DHA producer in the group.

## Data Availability

The original contributions presented in the study are included in the article; further inquiries can be directed to the corresponding authors.

## Supplementary Materials

The following supporting information can be downloaded at: https://www.mdpi.com/article/10.3390/md24010004/s1, Supplementary Table S1: Fatty acid (FA) composition of the examined sources as determined by gas chromatography–mass spectrometry (GC–MS). n-3, n-6, and n-9 denote omega-3, omega-6, and omega-9 fatty acids, respectively. Values represent the arithmetic mean ± standard deviation of triplicate measurements and are expressed as mg g^−1^ dry weight (DW).; Supplementary Figure S2: Spider web chart illustrating the relative distribution of individual fatty acids (FAs) in the examined omega-3 (n-3) sources, expressed as a percentage of the total FA content, as determined by gas chromatography–mass spectrometry (GC–MS). Supplementary Table S3: Glycerolipid content of the examined omega-3 sources determined by high-performance thin-layer chromatography (HPTLC) coupled densitometry. Values represent the arithmetic mean ± standard deviation of triplicate measurements and are expressed as mg g^−1^ dry weight (DW).
