# Supplementary material for: Omega-3 Source Matters: Comparative Lipid Signatures and Quantitative Distribution of EPA/DHA Across Marine Resources
Source: Mar Drugs. 2025 Dec 20;24(1):4. doi: 10.3390/md24010004 (PMC12843360; doi:10.3390/md24010004)
Supplement: Supplementary file 1 [file marinedrugs-24-00004-s001.zip › marinedrugs-4003549-supplementary/Supplementary Material/Supplementary Material 2.pdf]

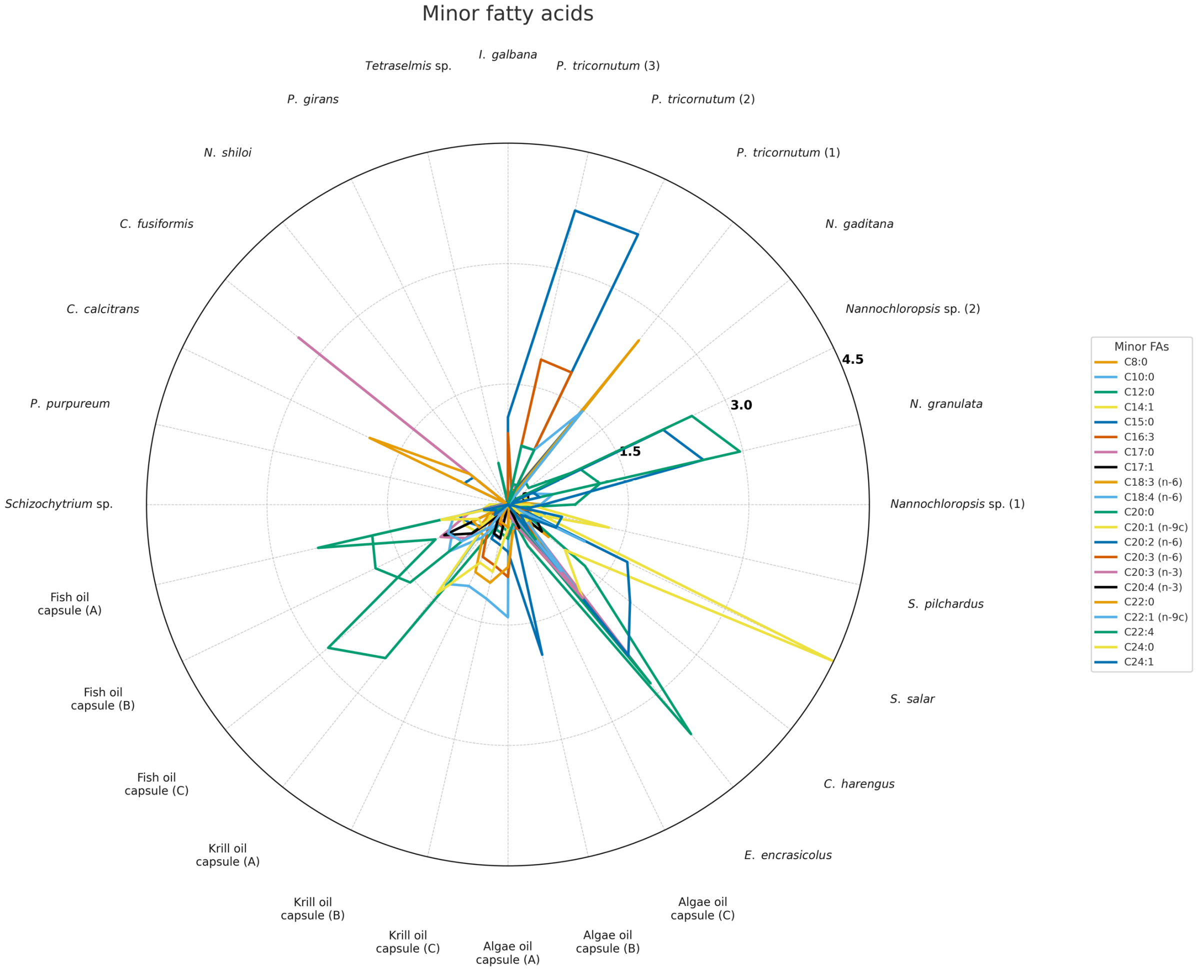

Supplementary Material 2. (S2) *Spider web chart illustrating the relative distribution of individual fatty acids (FAs) in the examined omega-3 (n-3) sources, expressed as a percentage of the total FA content, as determined by gas chromatography–mass spectrometry (GC–MS). Only FAs contributing less than 5% of the total FA content across all n-3 sources are displayed.*
