# Supplementary material for: Omega-3 Source Matters: Comparative Lipid Signatures and Quantitative Distribution of EPA/DHA Across Marine Resources
Source: Mar Drugs. 2025 Dec 20;24(1):4. doi: 10.3390/md24010004 (PMC12843360; doi:10.3390/md24010004)
Supplement: Supplementary file 1 [file marinedrugs-24-00004-s001.zip › marinedrugs-4003549-supplementary/Supplementary Material/Supplementary material legends .pdf]

Supplementary Material 1. (S1) Fatty acid (FA) composition of the examined sources as determined by gas chromatography–mass spectrometry (GC–MS). n-3, n-6, and n-9 denote omega-3, omega-6, and omega-9 fatty acids, respectively. Values represent the arithmetic mean ± standard deviation of triplicate measurements and are expressed as mg g^{-1} dry weight (DW).

Supplementary Material 2. **(S2)** Spider web chart illustrating the relative distribution of individual fatty acids (FAs) in the examined omega-3 (n-3) sources, expressed as a percentage of the total FA content, as determined by gas chromatography–mass spectrometry (GC–MS). Only FAs contributing less than 5% of the total FA content FA content across all n-3 sources are displayed.

Supplementary Material 3. (S3) Glycerolipid content of the examined omega-3 sources determined by high-performance thin-layer chromatography (HPTLC) coupled densitometry. Values represent the arithmetic mean ± standard deviation of triplicate measurements and are expressed as mg g^{-1} dry weight (DW).
